# The “Campaign of Education”

**Published:** 1900-11

**Authors:** 


### TEXAS MEDICAL JOURNAL.

AUSTIN, TEXAS.

A MONTHLY JOURNAL OF MEDICINE AND SURGERY.

EDITED AND PUBLISHED BY

F. E. DANIEL, M. D., AND S. E. HUDSON, M. D.

Published Monthly at Austin, Texas, by Drs. Daniel and Hudson. Subscription price $1.00 a year in advance.

Eastern Representative: John Guy Monihan, St. Paul Building, 220 Broadway, New York City.

Official organ of the West Texas Medical Association, the Houston District Medical Association, the Austin District Medical Society, the Brazos Valley Medical Association, the Galveston County Medical Society, and several others.

### The "Campaign of Education."

The committee appointed by the Texas State Medical Association at its annual meeting in Waco last April to compile, edit and publish the papers and discussions bearing on State medicine and public hygiene, and showing the necessity for medical and sanitary legislation, have performed that duty. Two thousand copies each of Dr. West's paper, "Maladministration of Public Medical Affairs in Texas," and the discussion on same; Dr. M. M. Smith's address, "The Public Health and the State's Duty to Protect It," and the discussion on it; and Dr. Harrison's paper and report as chairman of the Committee on a State Board of Health, together with a draft of a proposed bill, were printed by Von Boeckmann, Schutze & Co., Austin, Texas, each paper, with the discussion which followed its reading, being published separately in a neat pamphlet. A copy of each of the three pamphlets was mailed to every newspaper in Texas, and to every medical journal in the United States; also to each of the Representatives and Senators elected to the coming Legislature, to all the county and district judges, etc. Every newspaper was requested to either reproduce the matter or to make a liberal abstract of it and to publish it with such editorial comment as the importance of the subject warrants. The committee is composed of Dr. R. H. Harrison, Sr., Chairman, Columbus, Texas; Dr. F. E. Daniel, Austin; and Dr. J. B. Massie, Houston, Texas. Copies of the pamphlets may be had on request by persons who will "put them where they will do good."

A copy of each of these pamphlets has been mailed also to the members of the State Medical Association. The JOURNAL now urges the physicians throughout the State to lend their active and earnest co-operation to the promotion of the laudable object sought to be accomplished by the State Medical Association. Let each one make himself thoroughly acquainted with the subject, and then urge its importance upon the Representatives from his county; interest them in it, and enlist their support when the bill or bills come up in the Legislature. Every true physician should consider himself a committee of one, especially to work for reform in our medical and sanitary affairs, and to do all in his power to awaken a popular sentiment in favor of a *State Board of Health* in place of our primitive quarantine system. He should personally see the editor of his town or country paper, and induce him, through his columns, to make his readers aware of the neglect of the State to safeguard the public health; aware of the dangers lurking everywhere, and which, by the exercise of a little care, when they are thus brought to their attention, may be avoided. In every county let at least one newspaper ventilate the neglect and abuses discussed in these pamphlets, and enlist itself in the cause of reform. If the editor "hasn't the time" to write an editorial let some competent physician write it for him. If the paper cannot or will not reproduce the entire matter,—and it is too long to be printed in any but the big dailies—get the editor to abstract them, or do it for him. The State Medical Association wishes to get the matter to the attention of the *people*. When they understand the situation, and realize the danger lurking in every town and village, aye, in every household, by reason of unsanitary conditions—defective or no sewerage, foul privies, seepage wells, polluted streams, damp cellars, and above all, by reason of the *deadly doctor,* the uneducated fellow who is permitted by the great State of Texas to practice medicine by virtue of a "diploma" from some fake college—they will see the necessity of legislation for their own protection; and their Representative will respect their wishes or step down. A "diploma" is only evidence of a degree conferred (Webster) and conveys *no* rights or privileges whatever. It is *not a license,* but in Texas it is accepted as such. The right to license a person to exercise so grave a privilege as the practice of medicine belongs exclusively to the State; and for the State to permit any one to practice medicine without a license is a wrong inflicted upon the people. What right—moral or legal—have a party of unknown, perhaps unqualified, doctors in another State—perhaps, even, a lot of swindlers—to "license" a person to practice medicine in Texas? Or rather, what obligation is there upon our State authorities to recognize a diploma from any source as a license?

The Supreme Court of Texas has just decided that under our laws even one of those well-known fraudulent diplomas from Chicago —sold outright—is authority to practice. It is not even necessary that any district or other board (we have no State board) should "vise" it, or issue a certificate by reason of a diploma, as is done in some States. The holder of a "diploma" from anywhere, from any medical college, or so-called medical college "duly chartered in the State in which it is situated," is entirely independent of all boards; there is no supervision over the holder of a diploma from anywhere. All he has to do is to have his diploma "recorded" in the office of the county court where he desires to practice and the thing is done. He can then take life and tamper with health with impunity; he is a "legally qualified practitioner."

The "New York Medical College, at San Antonio—the charter for which was granted by the State of Texas and then annulled by the courts upon a petition of the Attorney-General at the instance of the medical profession—was an object lesson which, it is hoped, may yet prove a blessing, inasmuch as it will have emphasized the utter recklessness with which the State permits sharpers to practice medicine, and to license even negro porters to do the same after seventeen days alleged study; it is a *reductio ad absurdum,* and the occurrence has done more, we believe, to convince the Governor and the Attorney-General of the danger of such institutions and "diplomas" than all the efforts of the JOURNAL and of the State Medical Association in the last twenty years.

For the information of our readers whose support we are asking in this reform movement, and that they may be able to tell the Representatives-elect and the people just what is needed, we reproduce below a summary of the sanitary needs of the State, from an exhaustive editorial on the "State's Sin of Omission" which appeared in our January, 1899, issue:

"SANITARY NEEDS OF THE STATE.

"To administer quarantine, solely, Texas does not need a Board of Health. I maintain that that function can best be discharged by one man, provided he is a capable man, and that means a good deal. For when action is to be taken it should be prompt, and circumstances arise during the prevalence of epidemics which will not admit of delay for consultation.

But, as said elsewhere, quarantine is not all of sanitation, and Texas needs a Board of Health, or a Health Department, or a Commission, call it what you will, that shall be empowered and enabled, by a suitable appropriation, to institute and execute all the various measures for the prevention of disease and death, the better protection of the public health and the commerce of the State.

This subject is not understood by the people, nor by governors and legislators;—they have not given it thought.

For an understanding of the requirements, at this opportune time, when the medical profession will submit the needs of sanitary reforms to the Legislature, we may be pardoned for enumerating here, what is merely elementary knowledge with sanitarians, some of the principal functions pertaining to sanitation or preventive medicine:

Quarantine.—And it should be *rational,* and not absolute non-intercourse; and ample means for the purpose should be available.

Compilation and preservation of vital and mortuary statistics. Every birth should be recorded, and every death and the cause of it should also be recorded.

The unsanitary conditions of life and environment, so fruitful of disease and death, should be studied, and these can never be known and removed until we compile the vital statistics of the people.

Sanitary supervision of cities and towns, and of the dwellings and places of business of the people, factories and shops, schools, slaughter houses, etc.; of the slaughter of animals for food, and of the carcasses of such sold for food, as is the case even in Mexico. There every animal is passed upon by a government officer before it is killed, and the meat is stamped, numbered and a record kept of it.

The institution of drainage, the abolition of the cess-pool system in all communities; the protection of all streams and other sources of drinking water from pollution; the prevention of adulteration of food, etc.; the instruction of the people by publication, in the hygiene of living; the introduction of the general and special principles of sanitation in schools, prisons and factories; of compulsory vaccination, etc.; in short, the inculcation of such of the principles of sanitary science and the universal institution of such measures as will tend to lessen the death rate, and consequently add to the average length of life, and augment the material wealth and prosperity of the State."

